# Genetic differentiation of morphologically similar polyploid wheat species

**DOI:** 10.7717/peerj.20723

**Published:** 2026-03-03

**Authors:** Uzuk Kasymova, Asiye Ulug

**Affiliations:** Department of Biology, Kafkas University, Kars, Turkey

**Keywords:** Markers, Method development, Molecular diagnostic, Polyploid, Wheat barcoding

## Abstract

**Background:**

Wheat is a globally important polyploid crop, with hexaploid bread wheat (*Triticum aestivum* L.) and tetraploid durum wheat (*T. turgidum* subsp*. durum* (Desf.) Husn.) as its main cultivated forms. Despite distinct end-use properties, these species are morphologically similar, making their identification difficult. Traditional phenotypic approaches often fail to resolve closely related polyploid wheats, emphasizing the need for a reliable molecular diagnostic and wheat barcoding strategy.

**Method:**

This study developed and validated a multilocus molecular diagnostic framework for the discrimination of polyploid wheat species. The approach integrates plastid (*rbcL*, *matK*), nuclear ribosomal (*ITS2*, *IGS*), and nuclear-coding markers (*Glu-1* and *XDuPw167*), all amplified using the Polymerase Chain Reaction. Validation was performed using ten experimental samples and 203 reference sequences retrieved from the NCBI GenBank database. We developed and validated a multilocus molecular diagnostic method for the reliable discrimination of wheat species.

**Results:**

Plastid loci showed limited variation, whereas the *IGS* region contained a diagnostic 71 bp insertion linked to the D genome, clearly distinguishing hexaploids from tetraploids. The *Glu-1* and *XDuPw167* loci exhibited genome-specific polymorphisms that further differentiated the two species. The multilocus diagnostic method achieved over 95% amplification success and consistent sequence profiles across replicates, confirming its accuracy and reproducibility.

**Conclusions:**

The proposed molecular diagnostic method provides a reproducible, cost-effective, and high-resolution molecular diagnostic tool for reliable wheat species identification. By combining genome-specific nuclear and expressed sequence tag-simple sequence repeat (EST-SSR) markers, this approach establishes a robust and scalable system applicable to species authentication, seed purity testing, germplasm characterization, and genetic resource management.

## Introduction

The Neolithic transition to sedentary agricultural economies, which began approximately 13,000 years ago in the Fertile Crescent, led to the domestication of crops such as wheat and barley (Özdoğan, 2011; Broushaki et al., 2016; Feldman & Levy, 2023). Today, wheat (*Triticum* spp.) is cultivated worldwide and remains a cornerstone of global food security, with bread wheat (*T. aestivum* L*.*) and durum wheat (*T. turgidum* subsp. *durum* (Desf.) Husn*.*) as the two most economically important forms (Shewry & Hey, 2015). Despite their distinct end-use qualities—bread-making *versus* pasta production—these species are morphologically similar, making their reliable identification challenging.

Bread wheat, a hexaploid species (AABBDD), is characterized by a soft endosperm texture and highly extensible gluten that provides the elasticity and gas-holding capacity necessary for bread production. In contrast, tetraploid durum wheat (AABB) has a harder kernel texture and stronger, non-elastic gluten, which contributes to the firmness and cooking quality of pasta (Mastrangelo & Cattivelli, 2021). These biochemical and technological contrasts are driven largely by differences in glutenin subunit composition—particularly variation at the *Glu-1* loci—yet they are not consistently reflected in external morphology (Žilić et al., 2011; Langner et al., 2017; Roncallo et al., 2021). Both species exhibit similar spike and glume structures, and their grains overlap in size and color, leading to frequent misidentification, particularly in mixed, processed, or archaeological material (Ciftci et al., 2019; Feldman & Levy, 2023; Shan & Osborne, 2024). Thus, while phenotypic and agronomic traits underpin functional divergence, they alone are insufficient for precise taxonomic or practical discrimination.

Polyploidy has played a central role in the evolutionary history of wheat, with tetraploid *Triticum turgidum* L. (AABB) formed through hybridization of *Triticum urartu* Thumanjan ex Gandilyan (A genome) and *Aegilops speltoides* Tausch (B genome), and hexaploid *T. aestivum* (AABBDD) arising from hybridization between tetraploid wheat and *Aegilops tauschii* Coss. (D genome) (Levy & Feldman, 2022). While polyploidy provides genetic resilience and adaptability, it also results in morphological resemblance among species, complicating taxonomic classification and practical identification.

Traditional diagnostic approaches, including grain morphology or spike traits, often fail to distinguish bread wheat from durum wheat, especially in processed or mixed samples (Goriewa-Duba et al., 2018; Feldman & Levy, 2023). Similarly, widely used plastid barcodes such as *rbcL* and *matK* are highly conserved in wheat, limiting their utility for species discrimination (CBOL Plant Working Group, 2009; Hosein et al., 2017). Nuclear ribosomal markers such as *ITS2* provide somewhat greater resolution, but are subject to homogenization through concerted evolution, which reduces their discriminatory power in polyploid taxa (Álvarez & Wendel, 2003). Thus, there is a critical need for reliable, reproducible, and cost-effective molecular methods that can discriminate between wheat species.

This study addresses this gap by developing and validating a multilocus molecular approach for species authentication in polyploid wheats. The framework integrates plastid barcodes (*rbcL*, *matK*), nuclear ribosomal regions (*ITS2*, *IGS*), the *Glu-1* glutenin gene, and the expressed sequence tag (EST)-derived simple sequence repeat (SSR) marker *XDuPw167*. By combining genome-specific and functionally informative loci, the method enhances both universality and diagnostic precision, thereby filling a key methodological gap in the molecular characterization of polyploid crops. The multilocus method was evaluated using ten experimental samples and 203 reference sequences retrieved from GenBank, demonstrating accurate, reproducible, and cost-effective discrimination between *T. aestivum* and *T. turgidum* subsp. *durum*. Beyond improving species-level resolution, the proposed method offers practical advantages over existing molecular approaches: it provides higher discriminatory power than plastid barcodes, lower cost than genome-wide sequencing, and broad applicability to breeding, seed authentication, and germplasm management. Collectively, this study establishes a validated, reproducible, and accessible molecular diagnostic protocol for reliable wheat species identification, suitable for use in agricultural, industrial, and research contexts.

## Materials Methods

### Overview of the method

**Figure 1:**
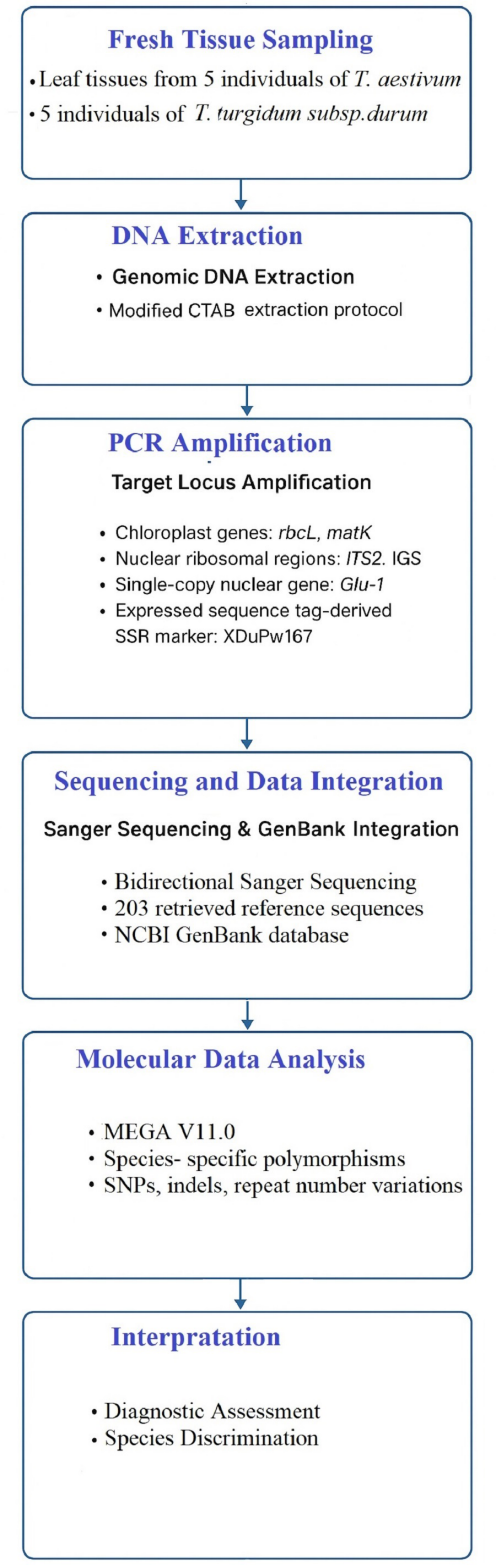
Methodological workflow for the molecular diagnosis of polyploid wheat species. Schematic overview of the multilocus PCR and sequencing approach combining plastid, nuclear ribosomal, and nuclear-coding markers (*rbcL*, *matK*, *IGS*, *Glu-D1*, *XDuPw167*) to discriminate between hexaploid and tetraploid wheat species.

This study develops and validates a multilocus molecular method for distinguishing polyploid wheat species (Fig. 1). The workflow consists of:

i. Plant material preparation and DNA extraction using a modified (cetyltrimethylammonium bromide) (CTAB) protocol.
ii. Polymerase Chain Reaction (PCR) amplification of standard plastid barcodes (*rbcL, matK*), nuclear ribosomal regions (*ITS2, IGS*), the *Glu-1* glutenin locus, and the EST-derived SSR marker *XDuPw167*.
iii. Sequencing and reference dataset integration by combining experimental data with publicly available GenBank sequences.
iv. Comparative sequence analysis to assess discriminatory power across loci, validate species-specific polymorphisms, and evaluate reproducibility of the method.

This workflow is designed to be reproducible in standard molecular biology laboratories without requiring high-cost next-generation sequencing platforms.

### Plant material and DNA extraction

Two representative wheat genotypes were selected for molecular analysis: *T. aestivum* cv. Gün 91 and *T. turgidum* subsp. *durum* cv. Selçuklu 97. Both cultivars are widely cultivated in Türkiye. For each genotype, five seeds were obtained from the Department of Field Crops, Faculty of Agriculture, Kırşehir Ahi Evran University, and used for DNA extraction and subsequent molecular analyses. The experimental design was aimed at validating the multilocus diagnostic method for species differentiation rather than assessing intra-varietal variability. Wheat seeds were germinated in pots under controlled conditions, and young leaves were collected ten days after germination. Leaf tissues were ground into a fine powder in liquid nitrogen using a sterile mortar and pestle. Genomic DNA was extracted from 15 mg of powdered tissue using a modified CTAB protocol (Doyle & Doyle, 1987) optimized for higher yield and purity. Briefly, samples were incubated in preheated extraction buffer (2% CTAB, 0.1 M Tris–HCl, pH 8.0, 20 mM EDTA, 1.4 M NaCl, and 1% PVP) at 65 °C for 2 h. After centrifugation at 13,000 rpm for 10 min, the supernatant was mixed with an equal volume of chloroform and centrifuged again at 13,000 rpm for 7 min. The aqueous phase was transferred to new tubes, and DNA was precipitated by adding 0.08 volumes of 7.5 M ammonium acetate and 0.54 volumes of isopropanol, followed by incubation at −20 °C for 2 h. The resulting DNA pellet was obtained by centrifugation at 14,000 rpm for 10 min, washed with 70% and 95% ethanol, air-dried, and dissolved in 100 µL of sterile nuclease-free water. DNA quality and concentration were assessed spectrophotometrically using A260/A230 and A260/A280 absorbance ratios. Detailed yield and purity data are presented in Table S1.

### Primers and PCR amplification

Specific primers were employed to amplify the plastid barcode loci (*rbcL, matK*), the nuclear ribosomal regions (*ITS2*, intergenic spacer (*IGS*)), and the EST-derived microsatellite locus *XDuPw167*, which is selected as one of the best EST-SSRs for wheat genotyping (Eujayl et al., 2002). In addition, short fragments of the *Glu-1* locus were targeted to assess ploidy-level variation in wheat. Since amplification with the primer pairs 52F and B52F did not yield a D-genome-specific product, additional primers were designed to amplify a region adjacent to the open reading frame of *Glu-1* (positions 209–130), as described by Fernandez et al. (2013). In the first-round PCR, fragments of approximately 132 bp were successfully amplified from the X- and Y-type glutenin subunits of the A, B, and D genomes. These products were not genome-specific: primer 52F amplified both A and D genomes, while primer B52F, which differs by a single base from 52F, amplified the B genome. To achieve genome specificity, nested PCR was performed. In these reactions, independent amplification of X- and Y-type subunits was required. Specifically, primers 52F and 156R (5′-ACCATGGCTGCGTGCAC-3′) amplified the Dx subunit (*Glu-1x*), whereas primers 52F and DY156R (5′-ACAATGGTTGTGTGCAC-3′) amplified the Dy subunit (*Glu-1y*), each yielding a fragment of 118 bp. The details of all primers and their respective amplification conditions are provided in Tables S2, S3. PCR reactions were carried out in final reaction volumes ranging from 23.5 to 25 µL containing 5 × HOT FIREPol^®^ Blend Master Mix (Solis BioDyne, Tartu, Estonia), and 20–30 ng of genomic DNA as template. The annealing temperatures and cycling conditions for each locus are summarized in Table S3 and were optimized individually to ensure specificity and amplification efficiency. For the *IGS* and *ITS2* loci, amplification was carried out at an annealing temperature of 60 °C for 30 cycles. The EST-SSR locus *XDuPw167* was amplified at 60 °C for 35 cycles, while plastid loci (*rbcL*, *matK*,) and *Glu-1* loci were amplified at an annealing temperature of 58 °C for 35 cycles. For each species, five biological samples were analyzed, and all PCR assays were performed in duplicate technical replicates to confirm the reproducibility of banding profiles and sequence data. Amplicons were resolved on 3% agarose gels stained with ethidium bromide and visualized under blue light (Vilber Lourmat, Collégien, France).

### Sequencing and data integration

To complement the experimental dataset and enhance the robustness of the analyses, 203 reference sequences representing the same loci in *T. aestivum* and *T. turgidum* subsp. *durum* were retrieved from the NCBI GenBank database. Sequence selection was based on verified species identification (*T. aestivum* or *T. turgidum* subsp. *durum*), complete coverage and the highest identity for the target locus, and the availability of full metadata, including accession number and source information. Both experimental and reference sequences were aligned and analyzed to assess interspecific and intraspecific variation. The amplified gene regions obtained from five seed samples of each wheat species were sequenced in both forward and reverse directions at BM Labosis (Ankara, Türkiye) using an ABI 310 Genetic Analyzer (PE Applied Biosystems) and an ABI 3730XL 96-capillary automated sequencer. Bidirectional sequencing minimized errors and resolved ambiguities, thereby ensuring high-quality chromatogram data. Sequence visualization, editing, alignment, and phylogenetic analyses were conducted using MEGA v11.0 software (Tamura, Stecher & Kumar, 2021). The NCBI BLASTn algorithm was employed to compare the obtained sequences with reference datasets and to identify highly similar sequences in GenBank (Johnson et al., 2008).

### Data analysis and validation of the method

Sequences were visualized, edited, and aligned using MEGA v11.0 (Tamura, Stecher & Kumar, 2021). BLASTn searches were performed against GenBank to confirm sequence identities.

Validation of the method involved:

Assessing discriminatory power of each locus (plastid *vs.* nuclear *vs.* EST-SSR).

Testing reproducibility across independent samples.

Comparing experimental results with public reference sequences.

Barcode regions (*rbcL, matK, ITS2*) served as baseline controls, representing current standard methods. The *IGS* region, *Glu-1* locus, and EST-SSR marker were evaluated as enhanced alternatives, with a focus on identifying loci that yield clear and reproducible discrimination between hexaploid and tetraploid wheat.

### Materials accessibility

Wheat seed material is available upon request from collaborating agricultural institutions. All primer sequences and PCR conditions are listed in Tables S2 and S3 to ensure reproducibility. Reference sequences are publicly accessible *via* GenBank, with accession numbers provided in Table 1. This transparency ensures that the method can be independently replicated and adapted by other researchers.

**Table 1:** BLAST results of *T. aestivum* and *T. turgidum* subsp. *durum* based on five gene regions. Samples yielding clean sequences were included in the analysis.

**Studied samples**	**Gene region**	**Base pair (bp)**	**Aligned species**	**GenBank accession code for the studied samples**	**Query coverage %**	**Percentage of identity %**	**Aligned sequences number retrieved from NCBI**
*T. aestivum*		872	*T. aestivum* (gb—MG958556.1)	PX171930	100	100	38
*T. turgidum* subsp. *durum*	*matK*	872	*T. turgidum* subsp. *durum* (gb—KU170120.1)	PX171931	100	100	5
*T. aestivum 1, 2, 3, 4, 5*	*rbcL*	603	*T. aestivum* (gb—MF597070.1)	PX171932–PX171936	100	100	38
*T. turgidum* subsp. *durum 1, 2, 3, 4, 5*		872	*T. turgidum* subsp. *durum* (gb—KM352501.1)	PX171937–PX171941	100	100	3
*T. aestivum 1, 4, 5*		195	*T. aestivum* (gb—KF482082.1)	PX138775	100	100	36
*T. aestivum 2*	*ITS2*	195	*T. aestivum* (gb—MF480405.1)	PX138776	99.49	100	
*T. aestivum 3*		195	*T. aestivum* (gb—MF480405.1)	PX138777	100	100	
*T. turgidum* subsp. *durum 1, 3, 4, 5*		195	*T. turgidum* subsp*. durum* (dbj—LC377262.1)	PX138780–PX138782, PX138783, PX138784	100	100	6
*T. turgidum* subsp. *durum 2*		195	*T. turgidum* subsp*. durum* (dbj—LC377262.1)	PX138781	100	99.49	
*T. aestivum 1, 3*		158	*T. aestivum* (gb—X07841.1)	PX207453, PX207455	100	95.51	5
*T. aestivum 2*	*IGS*	158	*T. spelta* (gb—AF147500)	PX207454	100	96.13	
*T. turgidum* subsp. *durum 1,2,3,4*		88	*Triticum turgidum* subsp. *durum* (emb—AJ009138.1)	PX207458, PX207459	100	91.95	3
*T. aestivum*	*XDupW167*	240	*T. aestivum* (gb—XM_044549473.1)	PX227498	99.17	97.92	1
*T. turgidum* subsp*. durum*		226	*T. dicoccoides* (gb—XM_037593326.1)	PX227499	94.17	92.50	1
*T. aestivum*	*Glu A1*	132	*T. aestivum* (emb—X61009.1)	PX171942	100	100	17
*T. turgidum* subsp*. durum*		132	*T. turgidum* subsp. *durum* (gb—JQ689002.1)	PX171943	100	100	1
*T. aestivum* 1, 2	*Glu B1*	132	*T. aestivum* (gb—FJ561336.1)	PX207448–PX207449	100	100	26
*T. turgidum* subsp *durum* 1, 2		132	*T. turgidum* subsp *durum* (gb—JQ689004.1)	PX207450–PX207451	100	100	3
*T. aestivum* 1,2,3	*Glu D1*	118	*T. aestivum* (gb—KM116496.1)	PX171944–PX171945–PX171946	100	98.31	20

## Results

### DNA extraction and amplification success

High-quality genomic DNA was successfully extracted from all wheat samples using the modified CTAB method, demonstrating the reliability and cost-effectiveness of this widely used protocol. PCR amplification was achieved for all six target loci (*rbcL*, *matK*, *ITS2*, *IGS*, *Glu-1*, *XDuPw167*); however, not all amplicons produced sequences of sufficient quality, and only high-quality reads were retained for subsequent BLASTn and comparative analyses. To enhance analytical robustness, an additional 203 sequences corresponding to the same loci were retrieved from the NCBI GenBank database and incorporated into the dataset. These reference sequences facilitated the detection of conserved motifs, diagnostic indels, single-nucleotide polymorphisms (SNPs), and repeat-number variations distinguishing the two species. Although the multilocus analyses consistently revealed diagnostic polymorphisms, overall sequence variation was extremely low both within and between species. Consequently, population-level statistics such as nucleotide diversity (*π*), genetic distance (*p*-distance), or fixation index (*Fst*) were not calculated, as they would lack biological relevance under such limited polymorphism. Instead, species differentiation was validated through fixed, reproducible sequence differences, confirmed by replicated amplifications showing over 95% success. Collectively, these results underscore the reliability and reproducibility of the multilocus diagnostic framework for distinguishing morphologically similar polyploid wheat species.

### Evaluation of barcode markers

The plastid regions *rbcL* and *matK,* as well as the nuclear *ITS2* locus, were amplified to establish a baseline for comparison with alternative markers. Three barcode regions, *matK, rbcL*, and *ITS2*, were amplified, yielding fragments of approximately 603 bp, 842 bp, and 195 bp in both wheat species, respectively (Table 1). Sequence analysis revealed that the *rbcL* region in five *T. aestivum* and five *T. turgidum* subsp. *durum* samples, as well as the *matK* region in one *T. aestivum* and one *T. turgidum* subsp. *durum* sample, were identical within each species. Furthermore, the studied *T. aestivum* and *T. turgidum* subsp. *durum* accessions showed 100% sequence similarity with 43 reference *rbcL* and 41 reference *matK* sequences of *T. aestivum* and *T. turgidum* subsp *durum* accessions previously deposited in the NCBI GenBank database (Table S4, Figs. S1, S2). These findings indicate that the barcode regions *rbcL* and *matK* showed complete conservation across *T. aestivum* and *T. turgidum* subsp. *durum*, and no polymorphism was detected within the dataset analyzed.

Amplification and sequencing of the *ITS2* region demonstrated a generally high level of sequence conservation between the studied *T. aestivum* and *T. turgidum* subsp. *durum* accessions. In studied *T. aestivum*, a limited number of nucleotide substitutions were observed at positions 164 and 175. These polymorphisms were not consistently detected across all individuals. By contrast, *T. turgidum* subsp. *durum* exhibited a highly conserved sequence profile, with only a single substitution identified at position 175 in one accession (*T. durum* 2). Comparative alignment with 40 reference *ITS2* sequences from GenBank confirmed the high sequence similarity of both wheat species with previously reported accessions, with only two nucleotide substitutions observed at positions 164 and 175 (Table 2, Fig. S3). Overall, the *ITS2* region exhibited low discriminatory power for distinguishing between *T. aestivum* and *T. turgidum* subsp. *durum*, although slight intra-specific variation was detected within *T. aestivum*.

**Table 2:** Comparison of the *ITS2* sequences obtained from samples of *T. aestivum*, *T. turgidum* subsp. *durum* and aligned sequences.

Species/Base Locations	**1**	** .**	**. **	**. **	**21**	**. **	** .**	**.**	**77**	**114**	**. **	**. **	**. **	**148**	**. **	**. **	**. **	**164**	**. **	**. **	**. **	**175**	**. **	**. **	**. **	**195**
*T. aestivum 1 *	**T**	**.**	**.**	**.**	**C**	**.**	**.**	**.**	**A**	**C**	**.**	**.**	**.**	**A**	**.**	**.**	**.**	**T**	**.**	**.**	**.**	**A**	**.**	**.**	**.**	**G**
*T. aestivum 2 *	**.**	**.**	**.**	**.**	**.**	**.**	**.**	**.**	**.**	**.**	**.**	**.**	**.**	**.**	**.**	**.**	**.**	**A**	**.**	**.**	**.**	**G**	**.**	**.**	**.**	**.**
*T. aestivum 3*	**.**	**.**	**.**	**.**	**.**	**.**	**.**	**.**	**.**	**.**	**.**	**.**	**.**	**.**	**.**	**.**	**.**	**.**	**.**	**.**	**.**	**G**	**.**	**.**	**.**	**.**
*T. aestivum 4*	**.**	**.**	**.**	**.**	**.**	**.**	**.**	**.**	**.**	**.**	**.**	**.**	**.**	**.**	**.**	**.**	**.**	**.**	**.**	**.**	**.**	**.**	**.**	**.**	**.**	**.**
*T. aestivum 5*	**.**	**.**	**.**	**.**	**.**	**.**	**.**	**.**	**.**	**.**	**.**	**.**	**.**	**.**	**.**	**.**	**.**	**.**	**.**	**.**	**.**	**.**	**.**	**.**	**.**	**.**
gb—KF482082.1*—T. aestivum*	**.**	**.**	**.**	**.**	**.**	**.**	**.**	**.**	**.**	**.**	**.**	**.**	**.**	**.**	**.**	**.**	**.**	**.**	**.**	**.**	**.**	**.**	**.**	**.**	**.**	**.**
gb—KC589709.1—*T. aestivum*	**.**	**.**	**.**	**.**	**.**	**.**	**.**	**.**	**.**	**.**	**.**	**.**	**.**	**.**	**.**	**.**	**.**	**.**	**.**	**.**	**.**	**.**	**.**	**.**	**.**	**.**
emb—FM998918.1—*T. aestivum*	**.**	**.**	**.**	**.**	**.**	**.**	**.**	**.**	**.**	**.**	**.**	**.**	**.**	**.**	**.**	**.**	**.**	**.**	**.**	**.**	**.**	**.**	**.**	**.**	**.**	**.**
gb—MF480405.1—*T. aestivum*	**.**	**.**	**.**	**.**	**.**	**.**	**.**	**.**	**.**	**.**	**.**	**.**	**.**	**.**	**.**	**.**	**.**	**.**	**.**	**.**	**.**	**G**	**.**	**.**	**.**	**.**
gb—OQ883696.1—*T. aestivum*	**.**	**.**	**.**	**.**	**.**	**.**	**.**	**.**	**.**	**.**	**.**	**.**	**.**	**.**	**.**	**.**	**.**	**.**	**.**	**.**	**.**	**G**	**.**	**.**	**.**	**.**
gb—PP814623.1—*T. aestivum*	**.**	**.**	**.**	**.**	**T**	**.**	**.**	**.**	**.**	**.**	**.**	**.**	**.**	**.**	**.**	**.**	**.**	**.**	**.**	**.**	**.**	**G**	**.**	**.**	**.**	**.**
*T. turgidum* subsp*. durum 1*	**.**	**.**	**.**	**.**	**.**	**.**	**.**	**.**	**.**	**.**	**.**	**.**	**.**	**.**	**.**	**.**	**.**	**.**	**.**	**.**	**.**	**.**	**.**	**.**	**.**	**.**
*T. turgidum* subsp*. durum 2*	**.**	**.**	**.**	**.**	**.**	**.**	**.**	**.**	**.**	**.**	**.**	**.**	**.**	**.**	**.**	**.**	**.**	**.**	**.**	**.**	**.**	**G**	**.**	**.**	**.**	**.**
*T. turgidum* subsp*. durum 3*	**.**	**.**	**.**	**.**	**.**	**.**	**.**	**.**	**.**	**.**	**.**	**.**	**.**	**.**	**.**	**.**	**.**	**.**	**.**	**.**	**.**	**.**	**.**	**.**	**.**	**.**
*T. turgidum* subsp*. durum 4*	**.**	**.**	**.**	**.**	**.**	**.**	**.**	**.**	**.**	**.**	**.**	**.**	**.**	**.**	**.**	**.**	**.**	**.**	**.**	**.**	**.**	**.**	**.**	**.**	**.**	**.**
*T. turgidum* subsp*. durum 5*	**.**	**.**	**.**	**.**	**.**	**.**	**.**	**.**	**.**	**.**	**.**	**.**	**.**	**.**	**.**	**.**	**.**	**.**	**.**	**.**	**.**	**.**	**.**	**.**	**.**	**.**
dbj—LC377262.1—*T. turgidum* subsp*. durum*	**.**	**.**	**.**	**.**	**.**	**.**	**.**	**.**	**.**	**.**	**.**	**.**	**.**	**.**	**.**	**.**	**.**	**.**	**.**	**.**	**.**	**.**	**.**	**.**	**.**	**.**
gb—KM352501.1—*T. turgidum* subsp *.durum*	**.**	**.**	**.**	**.**	**.**	**.**	**.**	**.**	**.**	**.**	**.**	**.**	**.**	**.**	**.**	**.**	**.**	**.**	**.**	**.**	**.**	**.**	**.**	**.**	**.**	**.**
gb—MZ230674.1—*T. turgidum* subsp*..durum*	**.**	**.**	**.**	**.**	**.**	**.**	**.**	**.**	**.**	**.**	**.**	**.**	**.**	**.**	**.**	**.**	**.**	**.**	**.**	**.**	**.**	**.**	**.**	**.**	**.**	**.**

### Validation of the *IGS* region as a ploidy diagnostic marker

**Figure 2:**
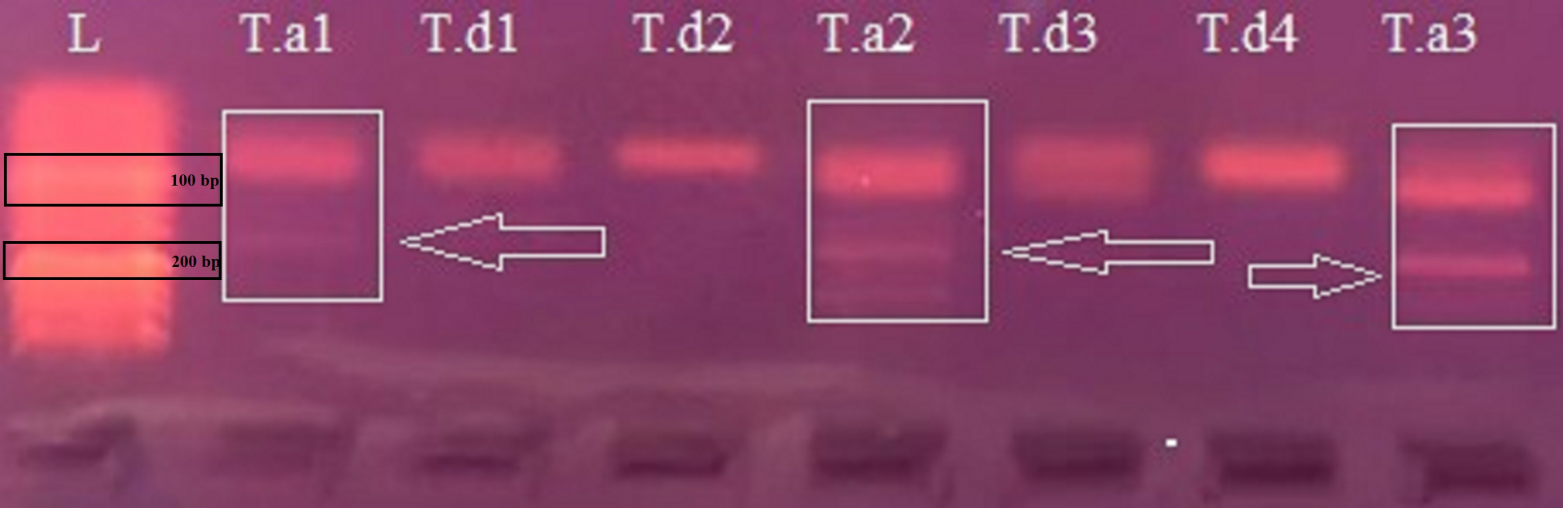
Amplification pattern of the IGS region in hexaploid and tetraploid wheat species. Amplification pattern of the IGS region in hexaploid *T. aestivum* and tetraploid *T. turgidum* subsp. *durum*. T.a, *T. aestivum*; T.d, *T. turgidum* subsp. *durum*; L, Low molecular weight DNA ladder.

**Figure 3:**
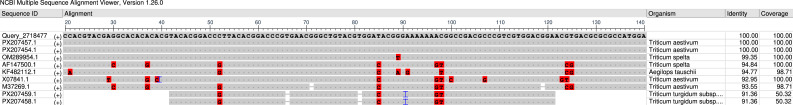
Multiple sequence alignment of the *IGS* region among the studied *T. aestivum* and *T. turgidum* subsp. durum samples and reference sequences of D-genome–containing species. Multiple sequence alignment of the IGS gene region comparing the studied *T. aestivum* sample, reference sequences of D-genome–containing species (*T. aestivum, T. spelta, A. tauschii)*, and the studied *T. turgidum* subsp. *durum* sample.

**Figure 4:**
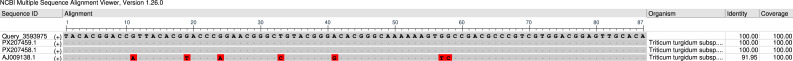
Pairwise sequence alignment of the *IGS* gene region between the studied *T. turgidum* subsp. *durum* sample and the *T. turgidum* subsp. *durum* reference sequence retrieved from NCBI.

Consistent amplification of the 87 bp A/B-genome fragment was obtained in all replicates, whereas the 155 bp D-genome–specific fragment appeared only under optimized PCR conditions and was detected in approximately 30% of reactions. Amplification and sequence alignment of the *IGS* region clearly distinguished hexaploid *T. aestivum* from tetraploid *T. turgidum* subsp. *durum*, yielding ∼155 bp products for *T. aestivum* (A, B, and D genomes) and ∼87 bp products for *T. durum* (A and B genomes only) (Fig. 2). Within the three studied *T. aestivum* samples, nucleotide polymorphisms were detected at one position (base 89) (Table S5). Alignment with reference sequences from *A. tauschii* (gb— KF482112.1)—the donor of the D genome—confirmed that the inserted region is homologous to D genome–specific sequences, further supporting the association of this insertion with the hexaploid genome composition. The *IGS* sequences of *Triticum spelta* L. (AF147500.1, OM289954.1), *T. aestivum* (X07841.1, M37269.1) and *A. tauschii* (KF482112.1), which possess the D genomes, were retrieved from the GenBank database and compared with the sequence data of the studied *T. aestivum* (Fig. 3). The *T. aestivum* sequences that matched the D genome from the wheat samples showed differentiation at 16 base positions. Four *T. turgidum* subsp. *durum* samples that have identical sequences exhibited the shorter structure without the insertion, consistent with their tetraploid genome composition. In the region shared with *T. aestivum* and *T. turgidum* subsp. *durum*, differentiation was observed at eight base positions in the 87 bp *IGS* region. Also, four deletions were detected in this shared region of the IGS gene. Only three alignments with reference sequences from *T. turgidum* subsp. *durum* (AJ009138.1, PX207458.1, PX207459.1) indicated seven nucleotide polymophisms (Fig. 4).

### *Glu-1* gene region: genome-specific polymorphism

In the present study, *Glu-1* subunits from the A, B, and D genomes were successfully amplified and analyzed in hexaploid bread wheat (*T. aestivum*) and tetraploid durum wheat (*T. turgidum* subsp. *durum*). First-round PCR generated 132 bp fragments corresponding to the *x*- and *y*-type glutenin subunits of the A and B genomes (Tables 1 and 3). These amplicons were not genome-specific and aligned with both Ax and Bx subunits. In contrast, nested PCR produced a 118 bp fragment specific to the Dx subunit, while the Dy subunit was not amplified under identical conditions (Tables 1 and 3). Comparative analysis of the *Glu-1* loci across subgenomes revealed that A and B genome sequences were identical between *T. aestivum* and *T. turgidum* subsp. *durum*, indicating intraspecific conservation. In contrast, the D genome, which is unique to hexaploid wheat, displayed clear sequence polymorphisms among *T. aestivum* accessions. Alignment with reference datasets (18 A, 29 B, and 20 D genome sequences from GenBank) confirmed the presence of both conserved and polymorphic sites. At the *Glu-A1.1x* locus, three variable sites were identified among 18 *T. aestivum* accessions, including one indel at position 29 and two SNPs. Additional substitutions were detected, such as a G insertion at position 48 and a T/C transition at position 71, confirmed by alignment with reference sequences (LT626214.1, DQ533690.1). In contrast, two *T. turgidum* subsp. *durum* accessions showed complete conservation at the same sites (Table 3, Fig. S4). Similarly, the *Glu-B1.1x* locus was highly conserved across both species. Among 28 *T. aestivum* and five *T. turgidum* subsp. *durum* accessions, only one variant was detected—a C substitution at position 44 in *T. aestivum* (AJ567973.1)—while all *T. durum* accessions were identical (Table 3, Fig. S5). By contrast, the *Glu-D1.1x* locus, found only in *T. aestivum*, exhibited significant sequence variability. Among 23 *T. aestivum* accessions, several SNPs were detected. Three accessions aligned with the reference sequence *T. aestivum* (KM116496.1) with 98.31% identity. Additional polymorphisms were identified, including a G substitution at position 44 (AB485591.1) and a T substitution at position 48 (KM116496.1), along with further accession-specific SNPs (Table 3, Fig. S6).

**Table 3:** Comparison of the *Glu 1* sequences obtained from samples of *T. aestivum*, *T. turgidum* subsp. *durum* and aligned sequences.

**Common sequence part of HMW- GS (** ***Glu 1*** **)**	**A, B, and D Genome**																		**A and B Genome**					
**HMW- GS (Glu A1.1)**	**1**	**11**	**29**	**33**	**44**	**48**	**65**	**70**	**71**	**73**	**82**	**87**	**93**	**100**	**109**	**112**	**118**	**119**		**.**	**126**	**.**	**132**	
**HMW- GS (Glu A1.1)**																								
*T. aestivum*	**A**	**G**	**G**	**T**	**A**	**T**	**C**	**C**	**A**	**A**	**T**	**C**	**A**	**A**	**G**	**G**	**G**	**T**		**.**	**T**	**.**	**C**	
emb—X61009.1—*T. aestivum*	**.**	**.**	**.**	**.**	**.**	**.**	**.**	**.**	**.**	**.**	**.**	**.**	**.**	**.**	**.**	**.**	**.**	**.**		**.**	**.**	**.**	**.**	
emb—LT626214.1—*T. aestivum*	**.**	**.**	**.**	**.**	**.**	**.**	**.**	**.**	**.**	**G**	**.**	**.**	**.**	**.**	**.**	**.**	**.**	**.**		**.**	**C**	**.**	**.**	
emb—LT626213.1*—T. aestivum*	**.**	**.**	**.**	**.**	**.**	**.**	**.**	**.**	**.**	**G**	**.**	**.**	**.**	**.**	**.**	**.**	**.**	**.**		**.**	**C**	**.**	**.**	
gb—DQ533690.1—*T. aestivum*	**.**	**.**	**-**	**.**	**.**	**.**	**.**	**T**	**.**	**.**	**.**	**.**	**.**	**.**	**.**	**.**	**.**	**.**		**.**	**.**	**.**	**.**	
*T. turgidum* subsp. *durum*	**.**	**.**	**.**	**.**	**.**	**.**	**.**	**.**	**.**	**.**	**.**	**.**	**.**	**.**	**.**	**.**	**.**	**.**		**.**	**.**	**.**	**.**	
gb—JQ689002.1—*T. turgidum* subsp*. durum*	**.**	**.**	**.**	**.**	**.**	**.**	**.**	**.**	**.**	**.**	**.**	**.**	**.**	**.**	**.**	**.**	**.**	**.**		**.**	**.**	**.**	**.**	
**HMW- GS (Glu B1.1)**	**A**	**A**	**G**	**T**	**C**	**T**	**C**	**C**	**A**	**G**	**A**	**C**	**T**	**G**	**A**	**A**	**T**	**C**		**.**	**C**	**.**	**C**	
*T. aestivum* 1	**.**	**.**	**.**	**.**	**.**	**.**	**.**	**.**	**.**	**.**	**.**	**.**	**.**	**.**	**.**	**.**	**.**	**.**		**.**	**.**	**.**	**.**	
*T. aestivum* 2	**.**	**.**	**.**	**.**	**.**	**.**	**.**	**.**	**.**	**.**	**.**	**.**	**.**	**.**	**.**	**.**	**.**	**.**		**.**	**.**	**.**	**.**	
emb—AJ567973.1—*T. aestivum*	**.**	**.**	**.**	**.**	**.**	**C**	**.**	**.**	**.**	**.**	**.**	**.**	**.**	**.**	**.**	**.**	**.**	**.**		**.**	**.**	**.**	**.**	
gb—KC254854.1—*T. aestivum*	**.**	**.**	**.**	**.**	**.**	**.**	**.**	**.**	**.**	**.**	**.**	**.**	**.**	**.**	**.**	**.**	**.**	**.**		**.**	**.**	**.**	**.**	
*T. turgidum* subsp. *durum* 1	**.**	**.**	**.**	**.**	**.**	**.**	**.**	**.**	**.**	**.**	**.**	**.**	**.**	**.**	**.**	**.**	**.**	**.**		**.**	**.**	**.**	**.**	
*T. turgidum* subsp*. durum* 2	**.**	**.**	**.**	**.**	**.**	**.**	**.**	**.**	**.**	**.**	**.**	**.**	**.**	**.**	**.**	**.**	**.**	**.**		**.**	**.**	**.**	**.**	
gb—JQ689004.1—*T. turgidum* subsp*. durum*	**.**	**.**	**.**	**.**	**.**	**.**	**.**	**.**	**.**	**.**	**.**	**.**	**.**	**.**	**.**	**.**	**.**	**.**		**.**	**.**	**.**	**.**	
**HMW- GS (Glu D1.1)**	**A**	**G**	**G**	**T**	**A**	**T**	**C**	**C**	**A**	**G**	**T**	**C**	**A**	**A**	**G**	**G**	**G**					
*T. aestivum 1*	**.**	**.**	**.**	**.**	**.**	**.**	**.**	**.**	**.**	**.**	**.**	**.**	**.**	**.**	**.**	**.**	**.**					
*T. aestivum* 2	**.**	**.**	**.**	**.**	**.**	**.**	**.**	**.**	**.**	**.**	**.**	**.**	**.**	**.**	**.**	**.**	**.**					
*T. aestivum* 3	**.**	**.**	**.**	**.**	**.**	**.**	**.**	**.**	**.**	**.**	**.**	**.**	**.**	**.**	**.**	**.**	**.**					
dbj—AB485591.1—*T. aestivum*	**.**	**.**	**.**	**.**	**G**	**.**	**.**	**.**	**G**	**.**	**.**	**.**	**.**	**.**	**.**	**.**	**.**					
gb—KM116496.1—*T. aestivum*	**.**	**.**	**.**	**.**	**G**	**.**	**T**	**.**	**G**	**.**	**.**	**.**	**.**	**.**	**.**	**.**	**.**					
emb—AJ577815.1—*T. aestivum*	**.**	**.**	**.**	**.**	**G**	**.**	**.**	**.**	**G**	**.**	**.**	**T**	**.**	**.**	**.**	**.**	**.**					

**Notes.**

The “_” and “.” symbols indicate the deletion and the identical bases, respectively.

### Variation in repeat number and sequence of *XDuPw167* Locus

**Figure 5:**
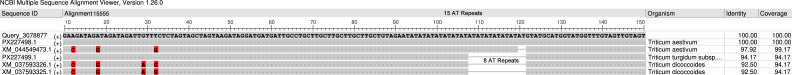
Sequence alignment of the *XDuPw167* EST-SSR region comparing the studied *T. aestivum* and *T. turgidum* subsp. *durum* samples with their corresponding reference sequences retrieved from NCBI.

The callose synthase gene spans 6,455 base pairs, within which the *XDuPw167* EST-SSR locus is located between positions 6,171 and 6,408 bp. Comparative sequence analysis of this gene between *T. aestivum* and *T. turgidum* subsp. *durum* revealed 14 single-nucleotide polymorphisms (SNPs) across the 6,455 bp region (Table 4). Notably, four SNPs were located within the EST-SSR locus (Fig. 5). Further sequence characterization of the *XDuPw167* EST-SSR region demonstrated clear genetic divergence between the studied hexaploid *T. aestivum* and tetraploid *T. turgidum* subsp. *durum*. The amplicon length of this locus was 240 bp in *T. aestivum* and 226 bp in *T. durum*. A distinct difference in the number of AT tandem repeats was also observed: *T. aestivum* harbored 15 repeats spanning positions 92–121, whereas *T. durum* contained only eight repeats distributed between positions 106–121 (Fig. 5). Alignment of the amplified *XDuPw167* sequences with GenBank references confirmed its association with the callose synthase gene. The *T. aestivum* sequence exhibited 100% identity and 97.92% similarity with the reference *T. aestivum* sequence (XM_044549473.1). Although no annotated callose synthase sequence is available for *T. turgidum* subsp. *durum*, its sequence aligned with tetraploid *T. dicoccoides*, showing 100% identity and 98.23% similarity (XM_037593326.1).

**Table 4:** Comparison of the β-1,3-glucan synthase sequence, including *XDuPw167* EST-SSR sequences of *T. aestivum* and *T. turgidum* subsp. *durum* retrieved from NCBI.

**β-1,3-glucan synthase**															***XDuPw167*** ** EST-SSR**																
**Sequence position**	**63**	**84-89**	**171**	**443**	**1826**	**2465**	**2468**	**2469**	**2471**	**2472**	**2473**	**2474**	**2489**	**5786**	**6268 - 6280**	**6281**	**6282**	**6283**	**6284**	**6285**	**6286**	**6287**	**6288**	**6289**	**6290**	**6291**	**6292**	**6293**	**6294**	**6295**	**6416**
*T. aestivum* XM 044549473.1	T	–	C	A	A	**T**	**C**	**T**	**T**	**G**	**A**	**T**	**T**	**T**	**ATATAT…**	**A**	**T**	**A**	**T**	**A**	**T**	**A**	**T**	**A**	**T**	**A**	**T**	**A**	**T**	**A**	**G**
*T. turgidum* subsp*. durum* XM 037593326.1	C	C	G	G	G	**C**	**A**	**C**	**A**	**C**	**C**	**A**	**C**	**C**	**—————**	**A**	**T**	**A**	**T**	**A**	**T**	**A**	**T**	**A**	**T**	**A**	**T**	**A**	**T**	**A**	**G**

**Notes.**

The “_” and “.” symbols indicate the deletion and the identical bases, respectively.

## Discussion

### Evaluation of barcode markers

This study utilized a focused experimental dataset consisting of ten samples (five per species). The primary objective was to establish and validate a reproducible multilocus molecular diagnostic rather than to explore intra-specific genetic diversity. While the dataset was adequate for methodological development and diagnostic testing, it was not intended to support population-level or geographic inferences. Sequence analyses of the chloroplast barcode regions (*rbcL* and *matK*) revealed complete sequence identity between *T. aestivum* and *T. turgidum* subsp. *durum*, consistent with previous reports highlighting the limited resolution of plastid barcodes in closely related polyploid crops (CBOL Plant Working Group, 2009; Chen et al., 2021). Consistent with our findings, Lubna-Asaf et al. (2022) reported that synteny analysis revealed high sequence similarity among *Triticum* plastomes, particularly within protein-coding regions such as *rbcL* and *matK*. The strong sequence conservation observed across accessions reflects both the slow evolutionary rate and functional constraint of chloroplast genes (Daniell et al., 2016), as well as the relatively recent divergence of hexaploid and tetraploid wheats (Wang et al., 2024). Similarly, Awad et al. (2017) demonstrated that the standard plastid barcodes *rbcL* and *matK* possessed limited discriminatory power among *Triticum* accessions in Egypt. Taken together, these studies and the present findings confirm that plastid loci such as *rbcL* and *matK* remain valuable as baseline markers for amplification consistency and higher-level phylogenetic inference but lack sufficient interspecific polymorphism for reliable species-level identification in polyploid wheat. Therefore, the inclusion of complementary nuclear markers is essential to achieve diagnostic resolution.

Analysis of the *ITS2* region revealed a high degree of sequence conservation between *T. aestivum* and *T. turgidum* subsp. *durum*. Although *ITS2* is one of the most widely used nuclear barcodes in plants due to its high copy number and strong discriminatory potential at the genus level (Álvarez & Wendel, 2003; CBOL Plant Working Group, 2009), it provided limited resolution in this study. Only minor polymorphisms were detected within *T. aestivum* (positions 164 and 175) and a single base substitution in one *T. turgidum* subsp. *durum* accession, which were insufficient for reliable species-level discrimination. The limited variability of *ITS2* in polyploid wheats likely reflects concerted evolution, a homogenizing process that reduces polymorphism among multiple ribosomal DNA repeats across subgenomes (Schoch et al., 2012; Wang et al., 2023). Additionally, the complex polyploid structure of wheat further constrains *ITS2* divergence by maintaining rDNA uniformity. Consistent with these findings, GenBank alignments also confirmed the conserved nature of *ITS2* sequences across accessions of both species. Overall, while *ITS2* remains valuable for broader phylogenetic and evolutionary studies, its limited sequence divergence restricts its diagnostic utility for distinguishing closely related polyploid wheat taxa.

The ribosomal DNA intergenic spacer (*rDNA IGS*) is a highly variable non-coding region located between the 26S and 18S rRNA genes and contains repetitive elements that evolve rapidly (Carvalho, Guedes-Pinto & Lima-Brito, 2011; Bendich & Rogers, 2023). Because of its high variability, the *IGS* region has been widely used to determine ploidy levels in wheat. Early studies demonstrated its diagnostic utility. Sallares, Allaby & Brown (1995) identified a 71 bp insertion specific to the D genome, allowing the distinction between tetraploid and hexaploid wheats in charred archaeological samples. Subsequent work has confirmed that *IGS*-derived polymorphisms accurately reflect genome composition, with expected PCR fragment sizes of approximately 158 bp for the D genome and 87 bp for the A/B genomes (Brown et al., 1998; Li et al., 2011; Oliveira et al., 2012; Değirmenci et al., 2022). In the present study, *IGS* analysis clearly demonstrated its diagnostic potential by differentiating tetraploid *T. turgidum* subsp. *durum* (A and B genomes) from hexaploid *T. aestivum* (A, B, and D genomes). The observed fragment sizes—approximately 87 bp for *T. turgidum* subsp. *durum* and 155 bp for *T. aestivum*—correspond to the absence or presence of the D-genome–specific insertion, respectively. This size variation provides a simple, reproducible, and cost-effective means for ploidy-level identification, making *IGS* particularly valuable when genome-wide sequencing is not feasible, such as in seed authentication, trade control, or archaeobotanical investigations.

Comparative sequence analyses further confirmed that the inserted *IGS* fragment in *T. aestivum* is homologous to *A.s tauschii*-derived sequences, supporting its established role as the D-genome donor (Mosulishvili et al., 2021; Dubcovsky & Dvořák, 2007). The consistent presence of this insertion in hexaploid species (*T. aestivum*, *T. spelta*) and its absence in tetraploid taxa (*T. durum*, *T. dicoccoides* (Asch. & Graebn.) Schweinf*.*) highlight the robustness of this marker for identifying ploidy levels. Base substitutions observed at different positions within the *IGS* region also reveal clear genomic differentiation between A, B, and D genomes. A total of 18 polymorphisms were detected within D-genome sequences and 19 substitutions between the A and B genomes, emphasizing the evolutionary divergence between tetraploid and hexaploid wheats (Levy & Feldman, 2022; Cavalet-Giorsa et al., 2024). These findings align with reports that structural differences in rDNA loci remain stable and diagnostic across wheat lineages (Huang et al., 2023; Cavalet-Giorsa et al., 2024). Collectively, these results confirm that the *IGS* region serves as a robust and informative molecular marker for both phylogenetic and diagnostic purposes. The presence or absence of the D-genome-specific insertion provides a straightforward sequence-based criterion that complements other nuclear and plastid markers in wheat systematics. Beyond its taxonomic value, the *IGS* marker also captures the genomic consequences of allopolyploidization, offering insights into the evolutionary history of wheat while providing a practical tool for breeding, germplasm management, and evolutionary studies.

### Nuclear marker performance

The *Glu-1* locus in wheat encodes high molecular weight (HMW) glutenin subunits, which are key determinants of dough elasticity and viscosity and, therefore play a central role in bread-making quality (Kroupina et al., 2023). This locus is located on the long arm of chromosome 1 in the A, B, and D genomes of wheat, with each genome harboring paralogous genes that encode two types of subunits: *x* and *y*. The *x* (132 bp) and *y* (132 bp) subunits form the major coding regions of glutenin proteins (Gu et al., 2006; Rathan et al., 2020). PCR amplification and sequencing of orthologous *Glu-1* regions across the A, B, and D genomes of hexaploid wheat (*T. aestivum*) and the A and B genomes of tetraploid wheat (*T. turgidum* subsp. *durum*) provided new insights into the genetic differentiation of polyploid wheat species. Among the three loci, *Glu-D1*, present only in hexaploid wheat, emerged as a key diagnostic marker whose sequence polymorphisms allow clear discrimination between tetraploid and hexaploid species. In contrast, *Glu-A1* and *Glu-B1* were highly conserved, confirming their limited discriminatory value and emphasizing the complementarity of conserved and variable loci within the multilocus framework. Amplification of Ax and Bx subunits from both species, and the Dx subunit exclusively from *T. aestivum*, confirmed the genome specificity of the markers and aligns with earlier studies describing the distinctive role of the D genome in hexaploid wheat (Fernandez et al., 2013; Gu et al., 2006). Comparative sequence analyses revealed strong conservation of *Glu-A1* and *Glu-B1* across both ploidy levels, with no nucleotide substitutions detected in *T. turgidum* subsp. *durum* and only minor indels and SNPs in *T. aestivum*. This stability reflects the evolutionary constraint and functional importance of these loci and explains the morphological similarity observed between bread and durum wheats despite genomic differences (Kroupina et al., 2023; Al-Khayri et al., 2023). By contrast, *Glu-D1* exhibited multiple polymorphisms, including clustered SNPs and accession-specific substitutions, consistent with the D genome’s origin from *Aegilops tauschii* and the introduction of novel allelic variation during allopolyploidization (Dvořák & Akhunov, 2005; Wang et al., 2024). Previous phylogenetic studies indicate that multiple tetraploidization events, involving divergent A genomes and less divergent B genomes, contributed to the complex genomic architecture of bread wheat (Gu et al., 2006; Orlovskaya et al., 2020; Levy & Feldman, 2022). The polymorphic nature of the Dx subunit therefore represents a valuable diagnostic feature, useful both for distinguishing ploidy levels and for breeding applications aimed at improving gluten quality (Zhang & Appels, 2024). Moreover, genome-wide SNP studies have reinforced the diagnostic and applied significance of such markers in wheat taxonomy and cultivar differentiation (Hyun et al., 2020; Winfield et al., 2016). From an evolutionary perspective, the contrasting patterns of conservation and polymorphism among *Glu-1* loci mirror the divergence history of the wheat genomes, which separated approximately 5–7 million years ago (Allaby, Banerjee & Brown, 1999; Zohary, Hopf & Weiss, 2012). The relatively slow evolutionary rate of the B genome (Feldman & Levy, 2023; Dvořák & Akhunov, 2005) explains its low variability, whereas the more recently incorporated D genome retains greater allelic diversity, providing a molecular signature of species differentiation. The *Glu-1* loci highlight both evolutionary stability and diagnostic potential in polyploid wheats. The conserved A and B genome loci reflect functional constraint, while the polymorphic *Glu-D1* locus represents a genome-specific marker of high diagnostic value and a reservoir of allelic diversity with direct implications for wheat breeding and improvement (Lee et al., 2025).

SSRs are well-established molecular markers due to their high informativeness and co-dominant inheritance. Expressed Sequence Tag–derived SSRs (EST-SSRs), in particular, offer the added advantage of being located within expressed genes, which makes them both evolutionarily conserved and transferable across related species (Eujayl et al., 2002; Yu et al., 2004; Wang et al., 2007). Despite their generally lower polymorphism compared with genomic SSRs, certain EST-SSR loci—such as *XDuPw167*—can display exceptional allelic variation and thus hold great diagnostic potential (Kong, Bai & Li, 2014; Manco et al., 2020). The *XDuPw167* locus, mapped to chromosome 6AL of wheat, is closely linked to the Sr13 resistance gene region. This locus corresponds to a genomic region annotated as encoding β-1,3-glucan synthase (callose synthase), an enzyme involved in the biosynthesis of callose—a β-1,3-glucan polysaccharide that serves as a structural component of the plant cell wall and accumulates during development and stress responses (Eujayl et al., 2002; Wang et al., 2015). In this study, the *XDuPw167* locus revealed distinct allelic differences between hexaploid *T. aestivum* and tetraploid *T. turgidum* subsp. *durum*. The hexaploid species produced a longer amplicon containing 15 repeat motifs, whereas the tetraploid carried only eight, indicating pronounced repeat-number variation. This sharp contrast aligns with the known dynamic nature of SSR evolution, likely driven by polymerase slippage within a non-coding segment of the β-1,3-glucan synthase gene (Sureshkumar et al., 2025). Because this gene encodes a vital enzyme for callose biosynthesis—a key component of plant defense—its flanking conserved coding regions maintain functionality while internal SSR regions accumulate neutral variation. This duality makes *XDuPw167* an ideal functional marker: highly polymorphic yet biologically meaningful (Nahas et al., 2020). Sequence comparisons further confirmed that the β-1,3-glucan synthase gene itself is highly conserved between the two wheat species, consistent with its essential role in cell wall integrity and stress response. However, the *XDuPw167* locus exhibited both repeat-number variation and SNPs, creating a dual polymorphic signature that enhances its diagnostic resolution. These polymorphisms likely occur in untranslated or intronic regions, or represent synonymous substitutions within the coding sequence, preserving protein function while reflecting genomic divergence (Castle, 2011). Functionally, β-1,3-glucan synthase mediates callose deposition during responses to biotic and abiotic stress (Seo et al., 2022). Therefore, variation at the *XDuPw167* locus may subtly influence defense-related gene expression, positioning this locus not only as a diagnostic marker but also as a functional marker with potential relevance for marker-assisted selection (MAS). Among all loci examined, *XDuPw167* demonstrated the strongest discriminatory power. Its combined repeat and SNP polymorphisms within a functional gene provide a unique molecular signature distinguishing tetraploid and hexaploid wheat while linking genetic variation to potential adaptive traits.

### Applied implications

The multilocus molecular framework developed in this study offers a reproducible, low-cost, and scalable strategy for discriminating morphologically similar polyploid wheat species. By integrating plastid, nuclear ribosomal, and nuclear-coding markers, the method maximizes both universality and diagnostic precision. The combined analysis of *IGS, Glu-1*, and *XDuPw167* loci provides complementary genetic information: *IGS* identifies ploidy-level differences (Huang et al., 2023), *Glu-1* reveals genome-specific polymorphisms (Lee et al., 2025), and *XDuPw167* delivers functional resolution within expressed genes (Nahas et al., 2020). This integrative approach ensures accurate species identification even when morphological characters or ecological data are inconclusive. Practically, the multilocus method holds significant potential for seed authentication, germplasm curation, and quality control in breeding programs. In applied settings, it can be adopted for rapid verification of wheat cultivar identity, detection of unintentional admixtures in seed lots, and monitoring of genetic integrity in breeding lines. Because the method relies on conventional PCR and Sanger sequencing rather than genome-scale analyses, it remains accessible to standard molecular laboratories without requiring high-cost sequencing platforms (Vasile et al., 2020; Satam et al., 2023). Recent genomic and taxonomic studies have emphasized the continued importance of classical locus-based assays for their reproducibility, cost efficiency, and compatibility with small or degraded DNA samples (Mahelka et al., 2021; Cavalet-Giorsa et al., 2024). Determining ploidy levels through genome-specific loci such as *IGS* and *Glu-D1* provides a faster and more economical alternative to flow cytometry or nuclear DNA quantification, which demand specialized equipment and fresh, high-quality tissue (Chang, Davis & Kwon-Chung, 2024; Nowicka et al., 2021). Beyond agricultural contexts, the framework has broader relevance for archaeobotanical and evolutionary studies, where ancient or degraded DNA often limits analytical options. The diagnostic power of the *IGS* region, in particular, enables reliable determination of wheat ploidy levels in archaeobotanical remains, contributing to reconstructions of early crop domestication and trade. Likewise, the genic SSR marker *XDuPw167* links molecular diversity to functional traits, offering opportunities for marker-assisted selection (MAS) and adaptive breeding.

## Conclusions

This study establishes a robust multilocus molecular framework for distinguishing morphologically similar polyploid wheat species. The integration of plastid, nuclear ribosomal, and nuclear-coding loci provides reliable species-level discrimination, with the *IGS*, *Glu-D*, and *XDuPw167* markers collectively capturing both structural and functional genomic variation. The multilocus approach offers high diagnostic accuracy, reproducibility, and cost efficiency, making it suitable for routine applications in seed authentication, germplasm management, and evolutionary research. These findings reinforce the continued relevance of targeted locus-based assays as accessible and practical tools complementing high-throughput genomic methods.

### Methodological conclusion

The validated multilocus method—combining *IGS, Glu-D*, and *XDuPw167* loci—constitutes a reproducible and scalable workflow for molecular identification of wheat species. Each marker provides complementary diagnostic information: *IGS* distinguishes ploidy levels, *Glu-D* reveals genome-specific variation, and *XDuPw167* detects functional polymorphisms within expressed genes. The protocol relies solely on standard PCR and Sanger sequencing, offering a rapid and low-cost alternative to genome-wide or cytogenetic analyses. Its modular design allows straightforward adaptation to other cereal taxa, supporting the broader development of standardized, locus-based diagnostic systems for complex polyploid crops.

##  Supplemental Information

10.7717/peerj.20723/supp-1 Supplemental Information 1 DNA extraction results, primer details, raw sequence data

10.7717/peerj.20723/supp-2 Supplemental Information 2 The sequence alignment between studied wheat species and retrieved sequences from NCBI

## Additional Information and Declarations

### DNA Deposition

The following information was supplied regarding the deposition of DNA sequences: The sequences are available at GenBank: PX171930, PX171931, PX171932–PX171936, PX171937–PX171941, PX138775, PX138776, PX138777, PX138780–PX138782, PX138783, PX138784, PX138781, PX207453, PX207455, PX207454, PX207458, PX207459, PX227498, PX227499, PX171942, PX171943, PX207448–PX207449, PX207450–PX207451, PX171944-PX171945-PX171946.

### Data Availability

The following information was supplied regarding data availability: The raw measurements are available in the Supplementary File.
