# Supplementary material for: Genetic differentiation of morphologically similar polyploid wheat species
Source: PeerJ. 2026 Mar 3;14:e20723. doi: 10.7717/peerj.20723 (PMC12965169; doi:10.7717/peerj.20723)
Supplement: Supplemental Information 1 [file peerj-14-20723-s001.docx]

**Genetic Differentiation of Morphologically Similar Hexaploid and Tetraploid Wheat Species**

**Supplementary Table 1.** DNA concentration and purity ratios

| **Species** | **DNA con. µg/ml** | **230/260 OD Ratio** | **260/280 OD Ratio** |
| --- | --- | --- | --- |
| *Triticum aestivum 1* | 213 | 1.97 | 1.82 |
| *Triticum aestivum 2* | 68 | 1.89 | 1.65 |
| *Triticum aestivum 3* | 124 | 2.14 | 1.98 |
| *Triticum aestivum 4* | 115 | 2.06 | 1.92 |
| *Triticum aestivum 5* | 142 | 2.15 | 1.99 |
| *Triticum turgidum* subsp*. durum 1* | 183 | 2.14 | 1.98 |
| *Triticum turgidum* subsp*. durum 1* | 225 | 2.06 | 1.92 |
| *Triticum turgidum* subsp. *durum 1* | 102 | 2.15 | 1.99 |
| *Triticum turgidum* subsp*. durum 1* | 150 | 2.00 | 1.85 |
| *Triticum turgidum* subsp*. durum 1* | 140 | 2.01 | 1.74 |

**Supplementary Table 2.** Primer details

| **Primer** | **5’-3’ Sequence** | **Product size (bp)** | **Reference** |
| --- | --- | --- | --- |
| *rbcL* | F:ATGTCACCACAAACAGAGACTAAAGC R:GTAAAATCAAGTCCACCRCG | 603 | CBOL Plant Working Group, 2009 |
| *IGS* | F:CGCCATGGAAAACTGGGCAA R:ACCTCTCGTACCCGTTCACGT | 87-158 | Fernandez et al., 2013 |
| *ITS2* | F:GCTGCGTTCTTCATCGATGC  R:GGAAGTAAAAGTCGTAACAAGG | 195 | White et al., 1990 |
| *matK* | F: CGTACAGTACTTTTGTGTTTACGAG  R: ACCCAGTCCATCTGGAAATCTTGGTTC | 800 | CBOL Plant Working Group, 2009 |
| *XDuPw167* | F: CGGAGCAAGGACGATAGG  R: CACCACACCAATCAGGAACC | 226-240 | Eujayl et al., 2002 |
| *52F* | F: ATTGCTCCTTGCTTATCCAGC  172 R: GGTGAAGGTTCAGGAC | 132 | Fernandez et al., 2013 |
| *B52F* | F: ATTGCTCCTTGCTTATCCAGC  172 R: GGTGAAGGTTCAGGAC | 132 | Fernandez et al., 2013 |
| *Dy* | F: ATTGCTCCTTGCTTATCCAGC  D156R: ACAATGGTTGTGTGCAC | 118 | Fernandez et al., 2013 |

**Suppmentary Table 3.** PCR conditions and cycling parameters for nuclear, ribosomal and chloroplast gene regions

| **Loci** | **Content** | **Volume (µL)** | **PCR Cyles** | | | |
| --- | --- | --- | --- | --- | --- | --- |
| ***IGS, ITS2*** | dH_2_O | 15.2 | First Denaturation 3 min | | | 95°C |
|  | Master mix | 4 | 30 cycles | | Denaturation 30 sec | 95°C |
|  | Primers(10µM) | 0.4+0.4 |  |  | Annealing 40 sec | 60°C |
|  | DNA (10ng/µL) | 5 |  |  | Extension 45 sec | 72°C |
|  | Average | 25 | Last extension 10min | | | 72°C |
| ***XDuPw167*** | dH_2_O | 13 | First Denaturation 3 min | | | 95°C |
|  | Master mix | 3.5 | 35 cycles | | Denatüration 1min | 94°C |
|  | Primers(10µM) | 0.4+ 0.4 |  |  | Annealing 1 min | 60°C |
|  | DNA (10ng/µL) | 5 |  |  | Extension 1 min | 72°C |
|  | Average | 23.5 | Last extension 10min | | | 72°C |
| ***rbcL, matK, 52F/172R***  ***52F/156R/Dy256R*** | dH_2_O | 13 | First Denaturation 3 min | | | 94°C |
|  | Master mix | 5 | 35 cycles | Denaturation 45 sec | | 94°C |
|  | Primers(10µM) | 0.5+0.5 |  | Annealing 50 sec | | 58°C |
|  | DNA (10ng/µL) | 5 |  | Extension 45 sec | | 72°C |
|  | Average | 24 | Last extension 10min | | | 72°C |

**Supplementary Table 4** Multiple sequence alignment of the *matK* and *rbcL* gene sequences from the studied *T. aestivum* and *T. turgidum* subsp. *durum*, together with retrieved sequences from NCBI

| **Species/Base Locations matK** | **1** | **.** | **.** | **.** | **304** | **.** | **.** | **.** | **310** | **311** | **.** | **.** | **.** | **424** | **.** | **.** | **.** | **451** | **.** | **.** | **.** | **495** | **.** | **.** | **498** | **.** | **.** | **.** | **814** | **.** | **.** | **872** |
| --- | --- | --- | --- | --- | --- | --- | --- | --- | --- | --- | --- | --- | --- | --- | --- | --- | --- | --- | --- | --- | --- | --- | --- | --- | --- | --- | --- | --- | --- | --- | --- | --- |
| *T. aestivum* | **T** | **.** | **.** | **.** | **C** | **.** | **.** | **.** | **T** | **G** | **.** | **.** | **.** | **G** | **.** | **.** | **.** | **C** | **.** | **.** | **.** | **T** | **.** | **.** | **T** | **.** | **.** | **.** | **A** | **.** | **.** | **G** |
| *gb\|MG958556.1\| T. aestivum* | **.** | **.** | **.** | **.** | **.** | **.** | **.** | **.** | **.** | **.** | **.** | **.** | **.** | **.** | **.** | **.** | **.** | **.** | **.** | **.** | **.** | **.** | **.** | **.** | **.** | **.** | **.** | **.** | **.** | **.** | **.** | **.** |
| *gb\|MG958554.1\| T. aestivum* | **.** | **.** | **.** | **.** | **.** | **.** | **.** | **.** | **.** | **.** | **.** | **.** | **.** | **.** | **.** | **.** | **.** | **.** | **.** | **.** | **.** | **.** | **.** | **.** | **.** | **.** | **.** | **.** | **.** | **.** | **.** | **.** |
| *gb\|MN977229.1\| T. aestivum* | **.** | **.** | **.** | **.** | **.** | **.** | **.** | **.** | **.** | **.** | **.** | **.** | **.** | **.** | **.** | **.** | **.** | **.** | **.** | **.** | **.** | **.** | **.** | **.** | **.** | **.** | **.** | **.** | **.** | **.** | **.** | **.** |
| *gb\|MN605258.1\| T. aestivum* | **.** | **.** | **.** | **.** | **.** | **.** | **.** | **.** | **.** | **.** | **.** | **.** | **.** | **.** | **.** | **.** | **.** | **.** | **.** | **.** | **.** | **.** | **.** | **.** | **.** | **.** | **.** | **.** | **.** | **.** | **.** | **.** |
| *gb\|AF164405.1\| T. aestivum* | **.** | **.** | **.** | **.** | **.** | **.** | **.** | **.** | **.** | **.** | **.** | **.** | **.** | **.** | **.** | **.** | **.** | **.** | **.** | **.** | **.** | **.** | **.** | **.** | **.** | **.** | **.** | **.** | **.** | **.** | **.** | **.** |
| *gb\|MN605257.1\| T. aestivum* | **.** | **.** | **.** | **.** | **.** | **.** | **.** | **.** | **.** | **.** | **.** | **.** | **.** | **.** | **.** | **.** | **.** | **.** | **.** | **.** | **.** | **.** | **.** | **.** | **.** | **.** | **.** | **.** | **.** | **.** | **.** | **.** |
| *ref\|XM 044543478.1\| T. aestivum* | **.** | **.** | **.** | **.** | **.** | **.** | **.** | **.** | **.** | **.** | **.** | **.** | **.** | **.** | **.** | **.** | **.** | **.** | **.** | **.** | **.** | **.** | **.** | **.** | **.** | **.** | **.** | **.** | **.** | **.** | **.** | **.** |
| *T. turgidum subsp durum* | **.** | **.** | **.** | **.** | **.** | **.** | **.** | **.** | **.** | **.** | **.** | **.** | **.** | **.** | **.** | **.** | **.** | **.** | **.** | **.** | **.** | **.** | **.** | **.** | **.** | **.** | **.** | **.** | **.** | **.** | **.** | **.** |
| *dbj\|LC377262.1\|T. turgidum subsp.durum* | **.** | **.** | **.** | **.** | **.** | **.** | **.** | **.** | **.** | **.** | **.** | **.** | **.** | **.** | **.** | **.** | **.** | **.** | **.** | **.** | **.** | **.** | **.** | **.** | **.** | **.** | **.** | **.** | **.** | **.** | **.** | **.** |
| *gb\|KU170120.1\|T. turgidum subsp.durum* | **.** | **.** | **.** | **.** | **.** | **.** | **.** | **.** | **.** | **.** | **.** | **.** | **.** | **.** | **.** | **.** | **.** | **.** | **.** | **.** | **.** | **.** | **.** | **.** | **.** | **.** | **.** | **.** | **.** | **.** | **.** | **.** |
| *gb\|KU170119.1\|T. turgidum subsp.durum* | **.** | **.** | **.** | **.** | **.** | **.** | **.** | **.** | **.** | **.** | **.** | **.** | **.** | **.** | **.** | **.** | **.** | **.** | **.** | **.** | **.** | **.** | **.** | **.** | **.** | **.** | **.** | **.** | **.** | **.** | **.** | **.** |

| **Species/Base Locations rbcL** | **1** | **.** | **.** | **.** | **80** | **.** | **.** | **.** | **130** | **.** | **.** | **.** | **211** | **.** | **.** | **.** | **300** | **.** | **.** | **.** | **420** | **.** | **.** | **.** | **501** | **.** | **.** | **.** | **603** |
| --- | --- | --- | --- | --- | --- | --- | --- | --- | --- | --- | --- | --- | --- | --- | --- | --- | --- | --- | --- | --- | --- | --- | --- | --- | --- | --- | --- | --- | --- |
| *T. aestivum 1* | **G** | **.** | **.** | **.** | **T** | **.** | **.** | **.** | **T** | **.** | **.** | **.** | **A** | **.** | **.** | **.** | **G** | **.** | **.** | **.** | **C** | **.** | **.** | **.** | **A** | **.** | **.** | **.** | **T** |
| *T. aestivum 2* | **.** | **.** | **.** | **.** | **.** | **.** | **.** | **.** | **.** | **.** | **.** | **.** | **.** | **.** | **.** | **.** | **.** | **.** | **.** | **.** | **.** | **.** | **.** | **.** | **.** | **.** | **.** | **.** | **.** |
| *T. aestivum 3* | **.** | **.** | **.** | **.** | **.** | **.** | **.** | **.** | **.** | **.** | **.** | **.** | **.** | **.** | **.** | **.** | **.** | **.** | **.** | **.** | **.** | **.** | **.** | **.** | **.** | **.** | **.** | **.** | **.** |
| *T. aestivum 4* | **.** | **.** | **.** | **.** | **.** | **.** | **.** | **.** | **.** | **.** | **.** | **.** | **.** | **.** | **.** | **.** | **.** | **.** | **.** | **.** | **.** | **.** | **.** | **.** | **.** | **.** | **.** | **.** | **.** |
| *T. aestivum 5* | **.** | **.** | **.** | **.** | **.** | **.** | **.** | **.** | **.** | **.** | **.** | **.** | **.** | **.** | **.** | **.** | **.** | **.** | **.** | **.** | **.** | **.** | **.** | **.** | **.** | **.** | **.** | **.** | **.** |
| *gb\|MF597070.1\|T. aestivum* | **.** | **.** | **.** | **.** | **.** | **.** | **.** | **.** | **.** | **.** | **.** | **.** | **.** | **.** | **.** | **.** | **.** | **.** | **.** | **.** | **.** | **.** | **.** | **.** | **.** | **.** | **.** | **.** | **.** |
| *gb\|MF597069.1\|T. aestivum* | **.** | **.** | **.** | **.** | **.** | **.** | **.** | **.** | **.** | **.** | **.** | **.** | **.** | **.** | **.** | **.** | **.** | **.** | **.** | **.** | **.** | **.** | **.** | **.** | **.** | **.** | **.** | **.** | **.** |
| *gb\|MG227483.1\| T.aestivum* | **.** | **.** | **.** | **.** | **.** | **.** | **.** | **.** | **.** | **.** | **.** | **.** | **.** | **.** | **.** | **.** | **.** | **.** | **.** | **.** | **.** | **.** | **.** | **.** | **.** | **.** | **.** | **.** | **.** |
| *T. turgidum subsp durum 1* | **.** | **.** | **.** | **.** | **.** | **.** | **.** | **.** | **.** | **.** | **.** | **.** | **.** | **.** | **.** | **.** | **.** | **.** | **.** | **.** | **.** | **.** | **.** | **.** | **.** | **.** | **.** | **.** | **.** |
| *T. turgidum subsp durum 2* | **.** | **.** | **.** | **.** | **.** | **.** | **.** | **.** | **.** | **.** | **.** | **.** | **.** | **.** | **.** | **.** | **.** | **.** | **.** | **.** | **.** | **.** | **.** | **.** | **.** | **.** | **.** | **.** | **.** |
| *T. turgidum subsp durum 3* | **.** | **.** | **.** | **.** | **.** | **.** | **.** | **.** | **.** | **.** | **.** | **.** | **.** | **.** | **.** | **.** | **.** | **.** | **.** | **.** | **.** | **.** | **.** | **.** | **.** | **.** | **.** | **.** | **.** |
| *T. turgidum subsp durum 4* | **.** | **.** | **.** | **.** | **.** | **.** | **.** | **.** | **.** | **.** | **.** | **.** | **.** | **.** | **.** | **.** | **.** | **.** | **.** | **.** | **.** | **.** | **.** | **.** | **.** | **.** | **.** | **.** | **.** |
| *T. turgidum subsp durum 5* | **.** | **.** | **.** | **.** | **.** | **.** | **.** | **.** | **.** | **.** | **.** | **.** | **.** | **.** | **.** | **.** | **.** | **.** | **.** | **.** | **.** | **.** | **.** | **.** | **.** | **.** | **.** | **.** | **.** |
| *dbj\|LC377262.1\|T.turgidum subsp durum* | **.** | **.** | **.** | **.** | **.** | **.** | **.** | **.** | **.** | **.** | **.** | **.** | **.** | **.** | **.** | **.** | **.** | **.** | **.** | **.** | **.** | **.** | **.** | **.** | **.** | **.** | **.** | **.** | **.** |
| *gb\|KM352501.1\|T. turgidum subsp.durum* | **.** | **.** | **.** | **.** | **.** | **.** | **.** | **.** | **.** | **.** | **.** | **.** | **.** | **.** | **.** | **.** | **.** | **.** | **.** | **.** | **.** | **.** | **.** | **.** | **.** | **.** | **.** | **.** | **.** |
| *gb\|MZ230674.1\|T.turgidum subsp durum* | **.** | **.** | **.** | **.** | **.** | **.** | **.** | **.** | **.** | **.** | **.** | **.** | **.** | **.** | **.** | **.** | **.** | **.** | **.** | **.** | **.** | **.** | **.** | **.** | **.** | **.** | **.** | **.** | **.** |

**Supplementary Table 5** Comparison of the *IGS* sequences obtained from the stduied *T. aestivum* samples

| **Species / Base Locations** | **Inserted part of D genome** | | | | | | | | |  | **Common sequence part of IGS for A, B and D Genome** | | | | | | | | | | | | | | | | | | | | |  | | **Inserted part of D genome** | | |
| --- | --- | --- | --- | --- | --- | --- | --- | --- | --- | --- | --- | --- | --- | --- | --- | --- | --- | --- | --- | --- | --- | --- | --- | --- | --- | --- | --- | --- | --- | --- | --- | --- | --- | --- | --- | --- |
|  | **1** | **29** | **30** | **34** | **37** | **38** | **39** | **40** | **41** | **42** | **52** | **60** | **65** | **66** | **75** | **81** | **84** | **85** | **89** | **91** | **98** | **99** | **100** | **101** | **108** | **123** | **124** | **125** | **126** | **127** | **130** | | **131** | | **137** | **156** |
| *T.aestivum 1* | T | A | G | C | A | C | A | C | G | T | C | G | G | T | T | T | A | T | G | - | A | C | G | G | C | A | C | G | T | G | G | | C | | T | C |
| *T. aestivum 2* | . | . | . | . | . | . | . | . | . | . | . | . | . | . | . | . | . | . | T | . | . | . | . | . | . | . | . | . | . | . | . | | . | | . | . |
| *T. aestivum 3* | . | . | . | . | . | . | . | . | . | . | . | . | . | . | . | . | . | . | . | . | . | . | . | . | . | . | . | . | . | . | . | | . | | . | . |


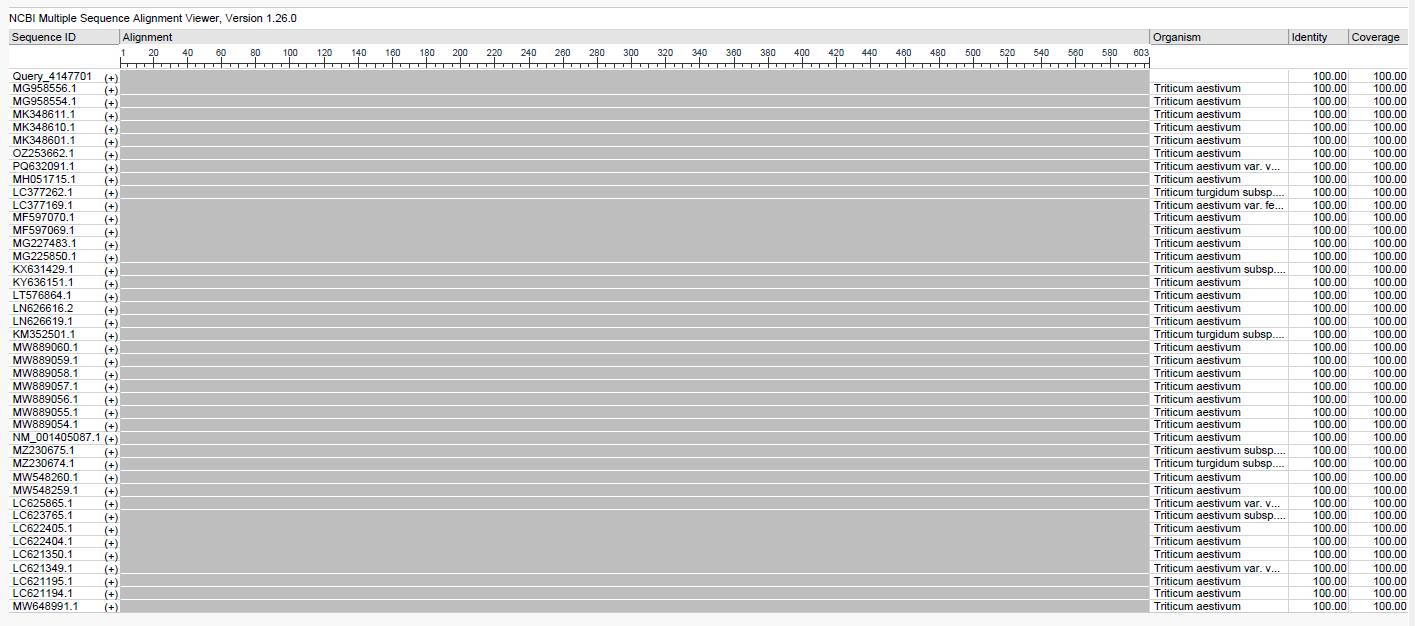


**Supplementary Figure 1.** Multiple sequence alignment of the *rbcL* gene region in T. aestivum and T. turgidum subsp. durum. The sequence query obtained in this study was identical across all analyzed T. aestivum and T. turgidum subsp. durum samples. Retrieved sequences from NCBI also showed no polymorphisms.


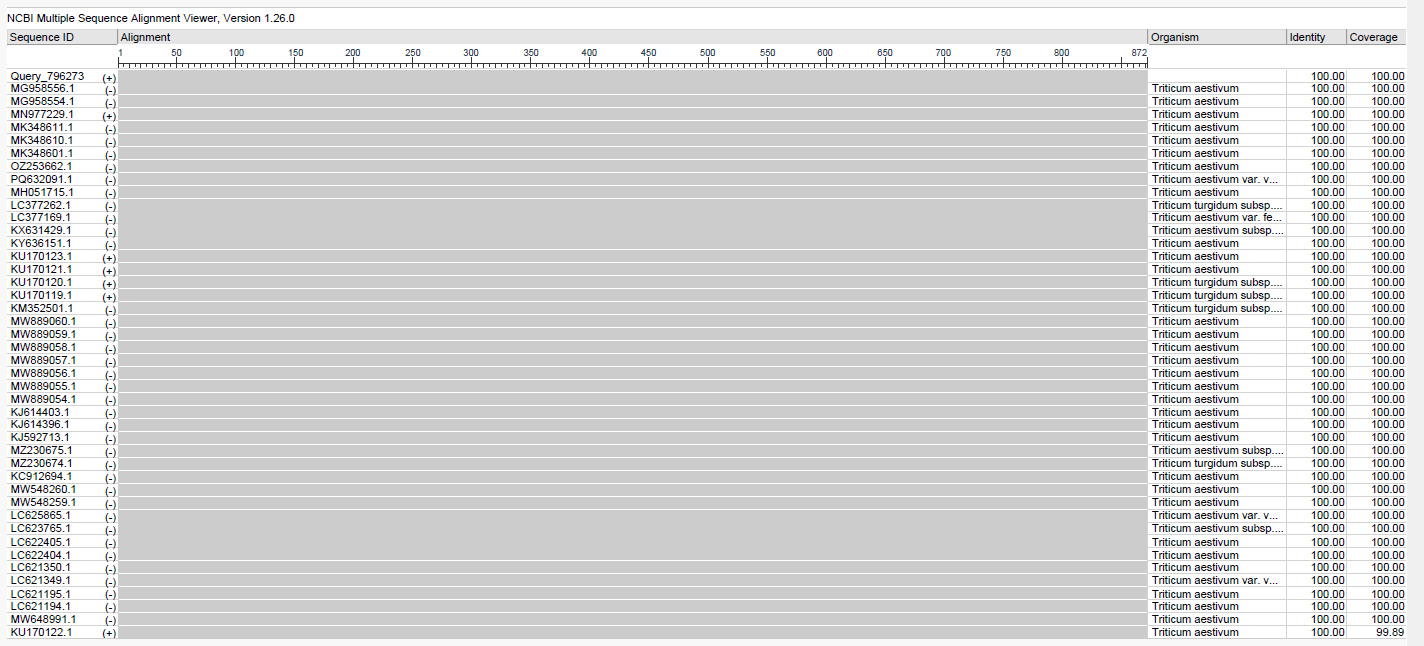


**Supplementary Figure 2.** Multiple sequence alignment of the *matK* gene region in T. aestivum and T. turgidum subsp. durum. The sequence query obtained in this study was identical across all analyzed T. aestivum and T. turgidum subsp. durum samples. Retrieved sequences from NCBI also showed no polymorphisms.


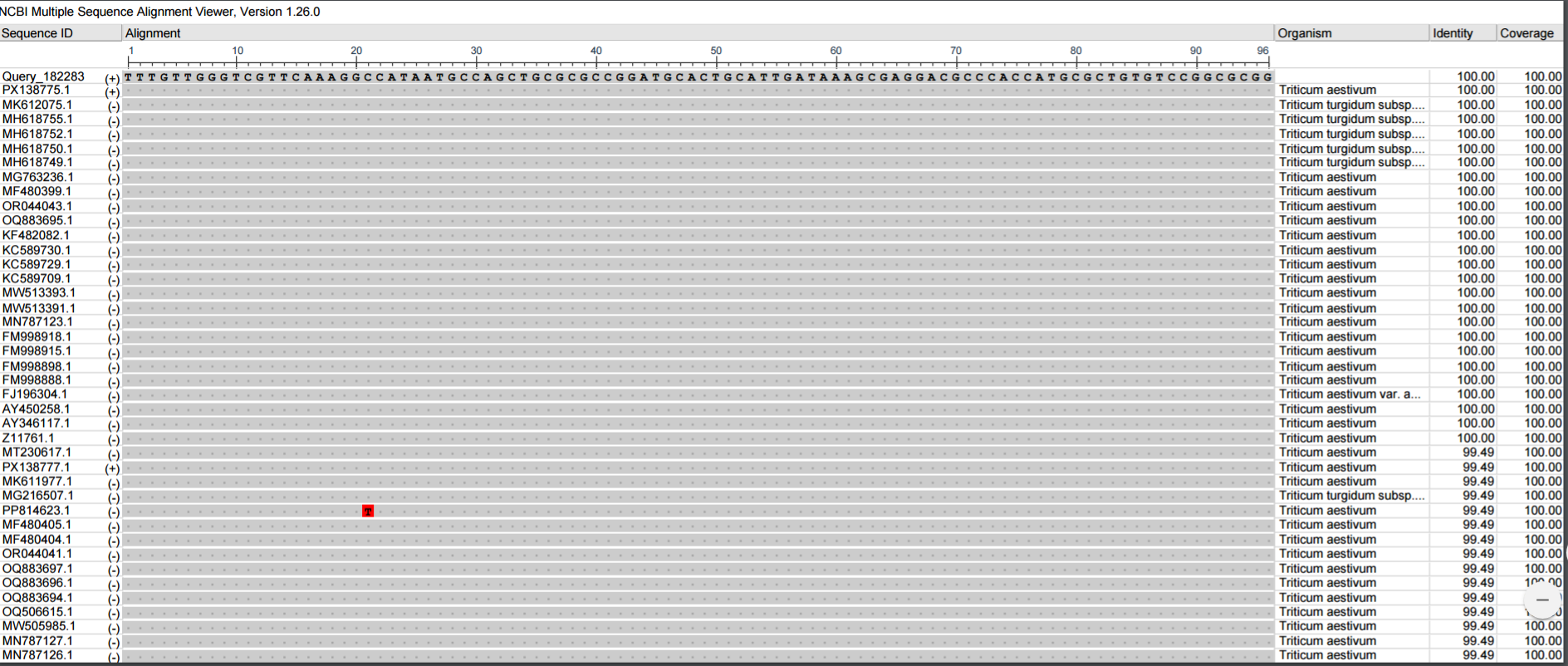


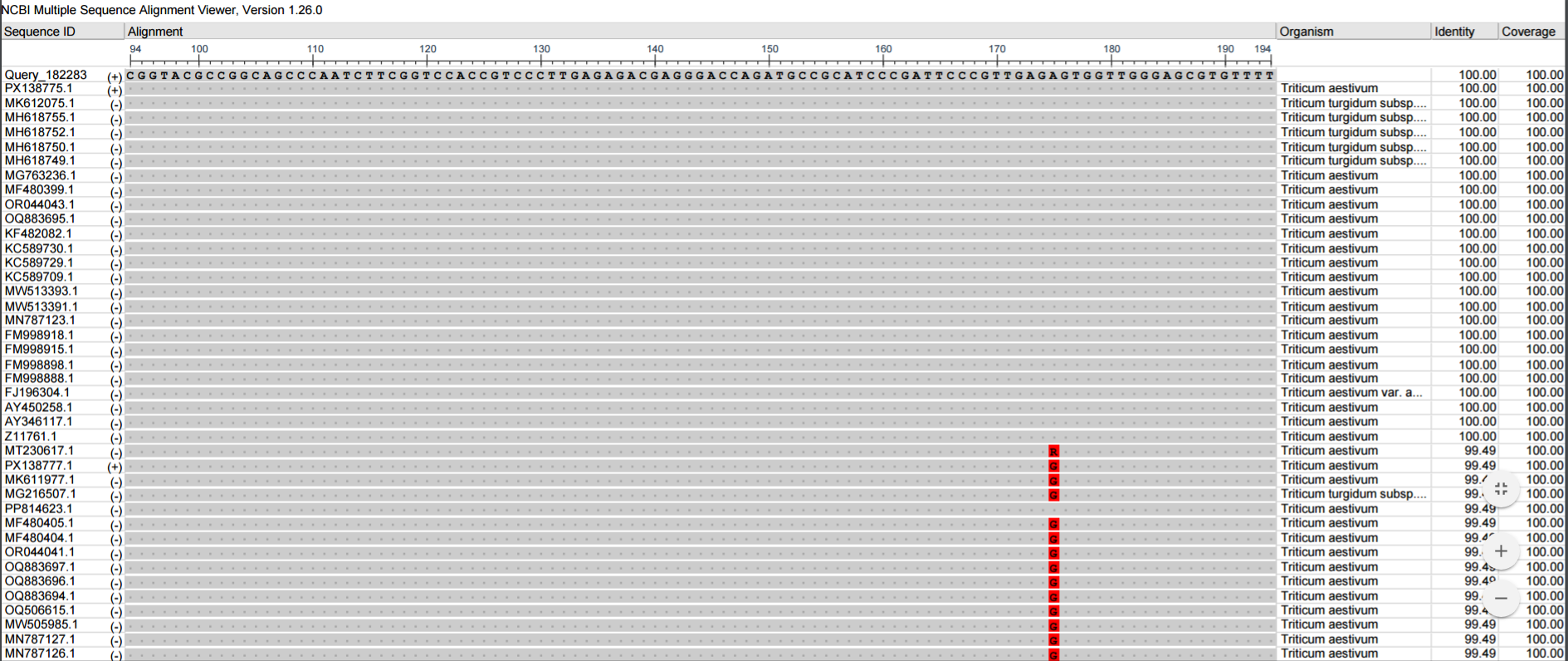


**Supplementary Figure 3.** Multiple sequence alignment of the *ITS2* gene region in *T. aestivum* and *T. turgidum* subsp. *durum*. The sequence query obtained in this study was identical for *T. aestivum* samples 1, 4, and 5, as well as for all *T. turgidum* subsp. *durum* samples. Retrieved sequences from NCBI revealed polymorphisms at two nucleotide positions (21 and 175 base positions).


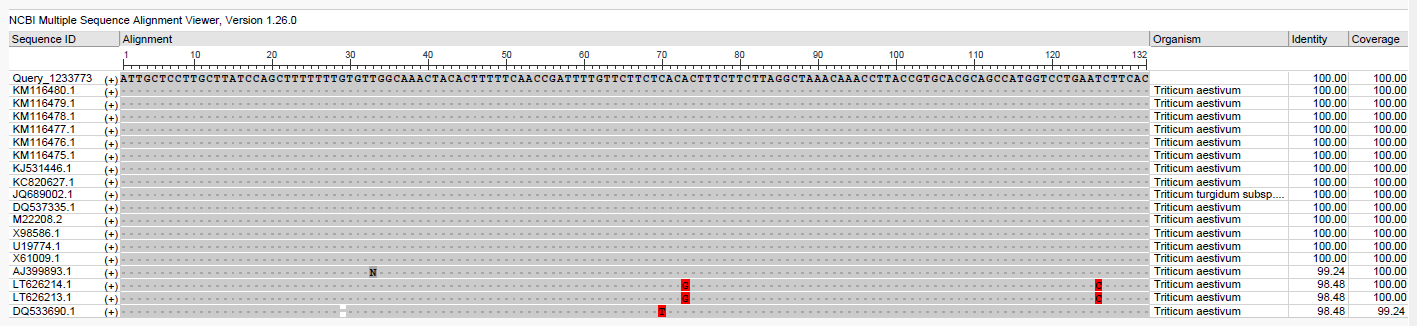


**Supplementary Figure 4.** Multiple sequence alignment of the HMW-GS (*Glu-A1.1*) region in *T. aestivum* and *T. turgidum* subsp. *durum*. The sequence query obtained in this study was identical across all analyzed *T. aestivum* and *T. turgidum* subsp. *durum* samples. Retrieved sequences from NCBI revealed polymorphisms at three nucleotide positions (69, 71, 126). There is one deletion at 29 bp position of DQ533690.1.


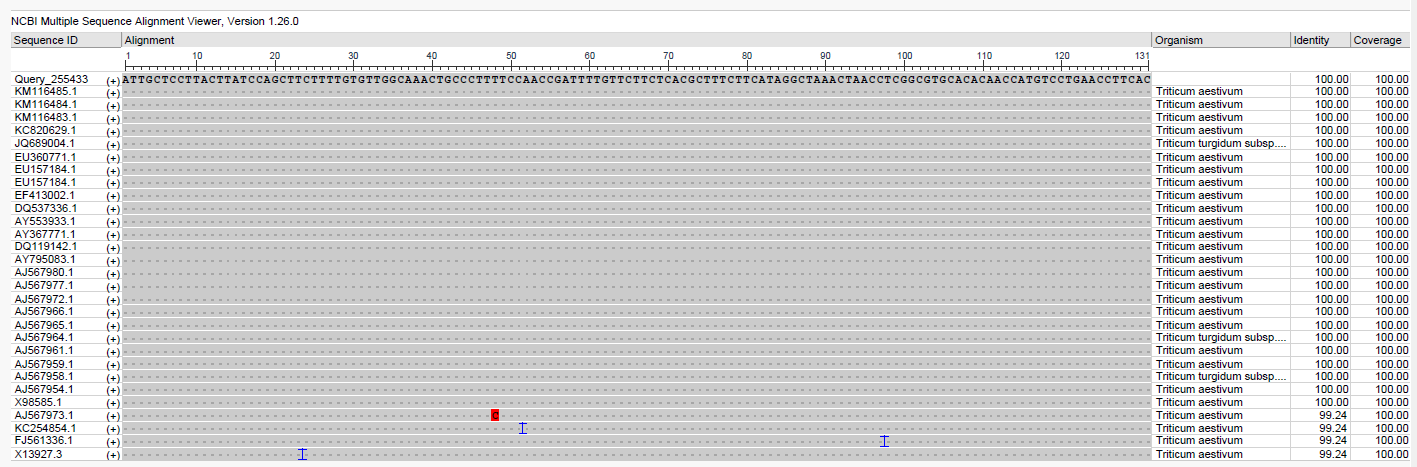


**Supplementary Figure 5** Multiple sequence alignment of the HMW-GS (*Glu-B1.1*) region in *T. aestivum* and *T. turgidum* subsp. *durum*. The sequence query obtained in this study was identical across all analyzed *T. aestivum* and *T. turgidum* subsp. *durum* samples. Retrieved sequences from NCBI revealed polymorphisms at one nucleotide position (48).


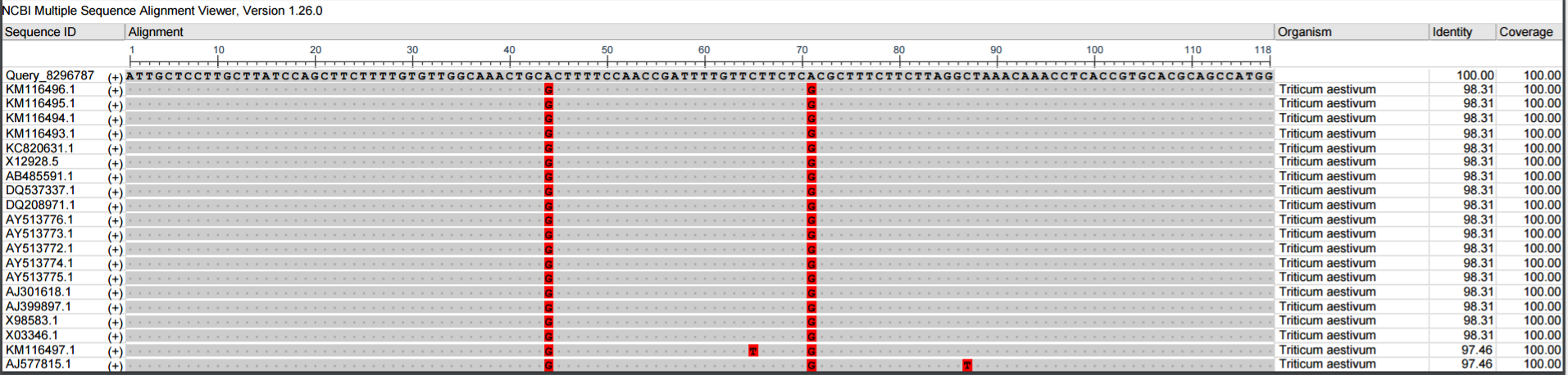


**Supplementary Figure 6** Multiple sequence alignment of the HMW-GS (*Glu-D1.1*) region in *T. aestivum*. The sequence query obtained in this study was identical across all analyzed *T. aestivum* samples. Retrieved sequences from NCBI revealed polymorphisms at four nucleotide positions (43, 65, 71, 87).
